# Vehicle detection in drone aerial views based on lightweight OSD-YOLOv10

**DOI:** 10.1038/s41598-025-09825-y

**Published:** 2025-07-11

**Authors:** Yang Zhang, Xiaobing Chen, Su Sun, Hongfeng You, Yuanyuan Wang, Jianchu Lin, Jiacheng Wang

**Affiliations:** 1 Huaiyin Institute of Technology, College of Computer and Software Engineering, Huaian, 223003 China; 2 Key Laboratory of Smart City and Virtual Reality of Jiangsu Province, Huaian, 223002 China; 3 Laboratory for Internet of Things and Mobile Internet Technology of Jiangsu Province, Huaian, 223001 China

**Keywords:** UAV, Object detection, OSD-YOLOv10, VisDrone-DET2019, UAVDT, Computational science, Computer science, Information technology

## Abstract

To address the challenges of low performance in vehicle image detection from UAV aerial imagery, difficulties in small target feature extraction, and the large parameter size of existing models, we propose the OSD-YOLOv10 algorithm, an enhanced version based on YOLOv10n. The proposed algorithm incorporates several key innovations: First, we employ online convolutional reparameterization to construct the OCRConv module and design a lightweight feature extraction structure, SPCC, to replace the conventional C2f module, thereby reducing computational load and parameter count. Second, we integrate an efficient dual-layer feed-forward hybrid attention module to enhance the model’s feature extraction capabilities. We also construct a dual small-target detection layer that combines shallow and ultra-shallow features to improve small-target detection. Finally, we introduce the DySample dynamic upsampling module to enhance feature fusion in the neck network from a point sampling perspective. Extensive experiments on the VisDrone-DET2019 and UAVDT datasets demonstrate that OSD-YOLOv10 achieves a 40.7% reduction in parameter count and a 3.6% decrease in floating-point operations, while improving accuracy and mean average precision by 1.3% and 1.6%, respectively. Compared to other YOLO series and lightweight models, OSD-YOLOv10 exhibits superior detection accuracy and lower computational complexity, achieving an optimal balance between high accuracy and low resource consumption. These advancements make it particularly suitable for deployment in UAV onboard hardware for vehicle target detection tasks. Code will be available online (https://github.com/Z76y/OSD-YOLO).

## Introduction

In recent years, unmanned aerial vehicles (UAVs) have been extensively deployed in agriculture^1^, traffic surveillance^2,3^, urban management^4^, and emergency response^5^ owing to their superior mobility, real-time data transmission capabilities, and efficient imaging performance. For road safety supervision, the integration of high-speed UAVs with object detection technology enables rapid vehicle identification and localization within road networks^6,7^, demonstrating significant application value for traffic flow statistics, safety monitoring, and vehicular positioning.Target detection in UAV aerial imagery is a core technological component, critically influencing both mission execution accuracy and the practical application’s efficacy. With advancements in deep learning, deep neural network-based object detection has emerged as the predominant solution for UAV vision systems. Current detection algorithms fall into two categories: Two-stage and one-stage detection algorithms. Two-stage detectors (e.g., R-CNN series^8–10^) excel in high-precision localization and small-object detection within complex scenarios through region proposal generation followed by refined classification and regression. However, their substantial computational demands and slow inference speeds hinder real-time deployment, particularly on embedded systems. Single-stage detectors (e.g., YOLO series^11–14^) reformulate detection as a regression problem, directly predicting object locations and categories from input images. This paradigm delivers marked speed advantages, making them ideal for the real-time processing of large-scale aerial imagery on embedded platforms^15,16^. They exhibit exceptional performance in intelligent transportation and disaster monitoring.

Nevertheless, fundamental limitations persist: Single-stage detectors exhibit severe accuracy degradation when processing small and densely clustered objects, particularly under long-range imaging or heavy occlusion in complex scenarios. More critically, UAV applications require extreme lightweight to accommodate the real-time processing of massive data streams on resource-constrained devices^17^. Consequently, ensuring high detection accuracy under stringent lightweight constraints has emerged as the core challenge constraining UAV object detection technology^18,19^. This irreconcilable accuracy-efficiency trade-off constitutes the primary research focus—where instantaneous scene interpretation and reliable information delivery directly determine mission outcomes in time-sensitive applications such as emergency response and environmental monitoring. Existing solutions remain incapable of simultaneously fulfilling the dual requirements for high timeliness and precision, significantly impairing mission-critical operations.

To address these challenges, researchers have focused on developing efficient and lightweight target detection networks. Recently, YOLOv10^14^ introduced an efficient architecture through innovations such as a lightweight classification head, spatial-channel decoupling downsampling, and rank-guided block design. Building on this, we propose OSD-YOLOv10, a lightweight UAV aerial target detection model based on YOLOv10. By significantly reducing the number of parameters and computational load, OSD-YOLOv10 achieves state-of-the-art detection speed while maintaining high accuracy, making it particularly suitable for vehicle target detection on UAVs with limited computational resources. The main contributions of this paper are as follows:

1. To address the issues of excessive parameter counts and computational load, we propose the OCRConv module and integrate it with a lightweight feature extraction structure, SPCC. Leveraging online convolutional reparameterization^20^ and partial convolution(PConv) operations^21^, this design achieves significant model lightweight without compromising performance.
2. To mitigate the problem of missed detections for small targets, we design an efficient spatial attention framework, Dual-Feed Mixed Attention (DFMA)Network, combined with a reconfigured dual-layer small-target detection network. This enhancement improves the model’s ability to recognize small targets in complex environments.
3. To address the loss of tiny target features during upsampling, we introduce DySample^22^, a dynamic upsampling module that enhances the feature fusion capability of the neck network from a point sampling perspective, ensuring better preservation of critical details.

Through these innovations, OSD-YOLOv10 achieves a balance between high accuracy and low resource consumption, making it a robust solution for real-time UAV-based vehicle target detection.

## Related work

### UAV **object detection**

Object detection is one of the core tasks in computer vision and serves as a crucial foundation for many advanced visual applications. With the increasing use of UAV (Unmanned Aerial Vehicle) technology in fields such as traffic monitoring^23,24^, disaster rescue^25^, and road planning^26^, object detection using UAV imagery has become a focal area of research. However, UAVs typically operate at altitudes ranging from tens to hundreds of meters, resulting in images where the targets are small in scale, low in resolution, and exhibit weak feature representation. These characteristics create significant technical challenges for effective object detection.

To solve the above problems, researchers have proposed various detection methods for UAV viewpoints.For instance, Song et al.^27^ based on the single-stage detection framework SSD, combined with the K-means + + clustering algorithm to optimize the anchor ratio, and introduced a low-pass filter to alleviate the aliasing effect caused by the pooling operation, thereby improving the recognition ability of small targets. Lu et al.^28^ designed a hybrid method of CNN and Transformer to achieve more effective UAV image detection by using multi-scale feature information.Hoanh et al.^29^ proposed a method of fusing the Transformer encoder-decoder and object focusing network to improve the performance of small object detection by optimizing the expression ability of multi-layer feature maps. Nguyen et al.^30^ designed a hybrid convolution-transformer architecture, which combined an adaptive attention mechanism and feature fusion module to strengthen fine-grained feature extraction and significantly enhance the robustness of small object recognition. Zhu et al.^31^ implemented TPH-YOLOv5, which replaces conventional prediction heads with Transformer-based structures and integrates channel-spatial attention mechanisms via CBAM modules, demonstrating superior performance in dense UAV scenarios. Zhang et al.^32^ proposed the PARE-YOLO algorithm, which involves the reconstruction of neck networks to enhance multi-scale feature extraction and fusion to improve the accuracy of UAV-based detection. Zhang et al.^33^ presented Drone-YOLO, which enhances YOLOv8’s detection capabilities for UAV imagery by utilizing RepVGG blocks as downsampling layers and integrating auxiliary feature branches within the neck network. In addition, Tahir et al.^34^ combined Swin Transformer and Soft-NMS mechanism to significantly improve the recognition ability of pedestrians and vehicles in occluded environments.

Although existing studies have achieved good performance in specific scenarios, they still have shortcomings in the balance between accuracy and computational efficiency, and more reasonable network design or optimization strategies are urgently needed to achieve better comprehensive performance.

### Network lightweighting techniques

The widespread use of deep learning models for various visual tasks has posed deployment challenges due to their large number of parameters and high computational costs, especially on resource-constrained edge devices. Therefore, network lightweighting has become a popular research topic in recent years. Current lightweighting techniques include lightweight network structure optimization, quantization^35^, knowledge distillation^36^, and network pruning^37^. Quantization lowers the precision of weights to reduce storage overhead and computational burden. Knowledge distillation uses high-performance teacher models to guide the learning process of lightweight student models, enabling them to retain key semantic information while achieving higher efficiency. Pruning aims to remove redundant connections or channels to compress model size and improve inference speed. However, these methods may lead to information loss, causing a decline in model performance. In contrast, network structure optimization designs efficient architectures for specific tasks and scenarios to minimize model size while maintaining high accuracy under limited computational resources. This approach is currently the most active area of research.In recent years, several innovative architectures have been proposed to achieve model lightweighting. For instance, Cao et al.^38^ utilized MobileNetV3 as the backbone network and incorporated depthwise separable convolution to control the number of channels, thereby significantly reducing both model parameters and computational load. Xu et al.^39^, introduced reparameterization techniques to decrease inference computation while preserving model accuracy. Zhang et al.^40^ employed partial convolutions to reconstruct the backbone and neck networks, minimizing redundant memory accesses. Wang et al.^41^ integrated the lightweight MobileNetV2 backbone network into the YOLOv4 framework to effectively compress the model size. Additionally, Wang et al.^42^ adopted Ghost convolution to construct a lightweight backbone network and combined it with the GSConv module to enhance feature representation capabilities, thus reducing computational complexity and improving the efficiency of target learning.

These methods serve as critical references for this study and guide the development of effective solutions. However, existing methods still exhibit limitations in balancing performance and efficiency. Some approaches overly prioritize real-time performance, sacrificing the integrity of network architecture design and leading to inadequate performance in complex scenarios. Other methods excessively focus on accuracy, resulting in an initially excessive number of parameters. Even after lightweight processing, these models often fail to meet the requirements for embedded deployment. Improving accuracy and generalization capabilities while preserving the lightweight characteristics of the model remains a pivotal challenge in the field of lightweight UAV target detection.

## Methodology

### YOLOv10

The YOLOv10 model represents the most recent progress in the YOLO family of object detection algorithms and has gained widespread attention in the global research community. YOLOv10^14^ introduces a unified dual assignment strategy, eliminating the reliance on non-maximum suppression (NMS) during training, a first in the YOLO series. Additionally, YOLOv10 adopts a model design strategy driven by overall efficiency and accuracy, incorporating innovations such as lightweight classification heads, spatial-channel decoupled downsampling, and a rank-guided block design. These advancements enable YOLOv10 to achieve outstanding performance on the COCO dataset, maintaining high detection accuracy in complex backgrounds while remaining lightweight enough for deployment on embedded devices.

YOLOv10 offers six variants: YOLOv10n, YOLOv10s, YOLOv10m, YOLOv10b, YOLOv10l, and YOLOv10x. Given the stringent requirements of UAV applications for model efficiency and lightweight design, this paper selects YOLOv10n as the baseline model to evaluate its performance in vehicle and pedestrian detection for UAV aerial imagery. YOLOv10n comprises four main components: Input, Backbone, Neck, and Head.

**Input: **Handles image enhancement tasks, including HSV augmentation, image translation, scaling, flipping, and mosaic augmentation.

**Backbone**: Builds upon the C2f and SPPF structures from YOLOv8 and introduces three novel modules: SCDown, C2fCIB, and PSA. SCDown achieves spatial-channel decoupled downsampling by combining pointwise and depthwise convolutions. C2fCIB, the core module of YOLOv10, employs depthwise convolution for spatial mixing and pointwise convolution for efficient channel mixing within a compact inverted block structure. To address the high computational cost of self-attention mechanisms, YOLOv10 integrates a partial self-attention mechanism called Position-wise Spatial Attention (PSA), enhancing model performance.

**Neck: **Retains the FPN and PANet structures for effective multi-scale feature fusion.

**Head: **Utilizes lightweight One-to-one Head and One-to-many Head detection heads during training, with final detection results generated through the unified dual assignment strategy.

Through these innovative designs, YOLOv10 significantly improves computational efficiency while maintaining high detection accuracy, making it well-suited for deployment on edge devices in UAV applications.

### OSD-YOLOv10

**Fig. 1:**
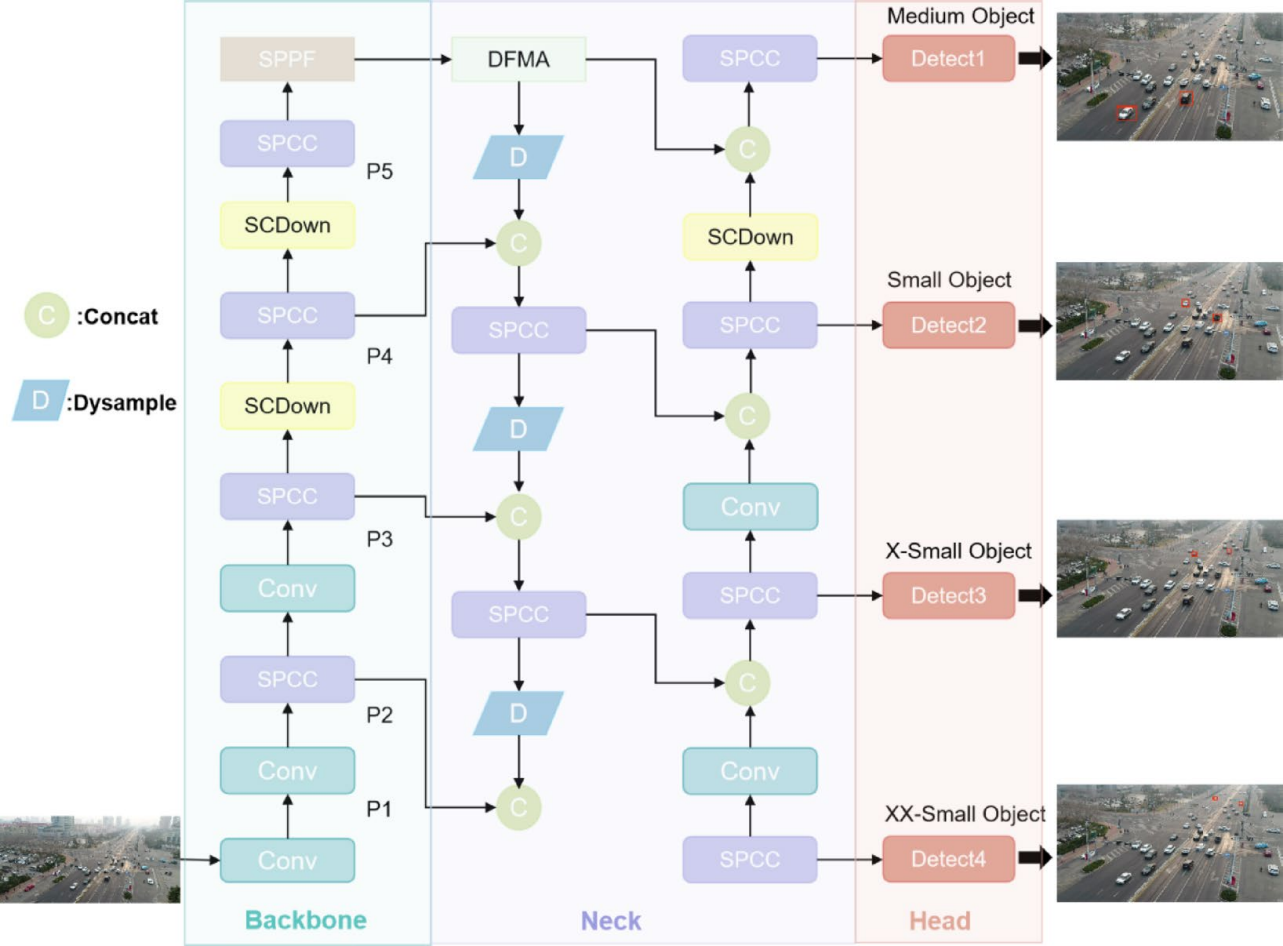
Structure of OSD-YOLOv10(compared with YOLOv10, Conv is mainly replaced by OCRConv, C2f is replaced by SPCC designed in this paper, upsampling is replaced by Dysample, PSA is replaced by DFMA designed in this paper and combine reconstruct the small object layer). (The model diagram was created by our collaborator, Yang Zhang. All images displayed are from the publicly available VisDrone2019 dataset.)

This paper proposes a novel and efficient lightweight vehicle target detection model, OSD-YOLOv10, designed for UAV applications, as illustrated in Fig. 1. The model incorporates several key innovations to enhance performance while maintaining computational efficiency. First, to reduce computational complexity, the original C2f module in YOLOv10 is replaced with an integrated lightweight SPCC network. This modification not only reduces model complexity but also enables more efficient multi-scale feature extraction and detection. Additionally, standard convolutions are partially replaced with the efficient OCRConv module, which enhances the model’s multi-scale feature extraction capabilities for UAV imagery while keeping computational costs low, thereby improving overall performance. Second, to address the challenge of small target detection, an efficient Dual-Feed Mixed Attention (DFMA)network mechanism is introduced at the neck of the network. This module enhances the model’s ability to learn global information from input feature maps, thereby improving the network’s expressive power. Furthermore, a dual small target detection layer is constructed in the head of the network. This layer combines a shallow small target detection layer, which fuses features from the P2 layer, with a small target detection layer that integrates the shallowest features from the P1 layer. This dual-layer design significantly enhances the model’s accuracy in detecting small targets. Finally, to minimize the loss of target features during upsampling, the DySample module is introduced. In dense scenes where target feature information is highly overlapping, DySample’s adaptive sampling position weight generation mechanism preserves more complete details across hierarchical feature maps, ensuring robust feature representation. The detailed design principles and implementation of each module in OSD-YOLOv10 are elaborated in the following sections.

### OCRConv

**Fig. 2:**
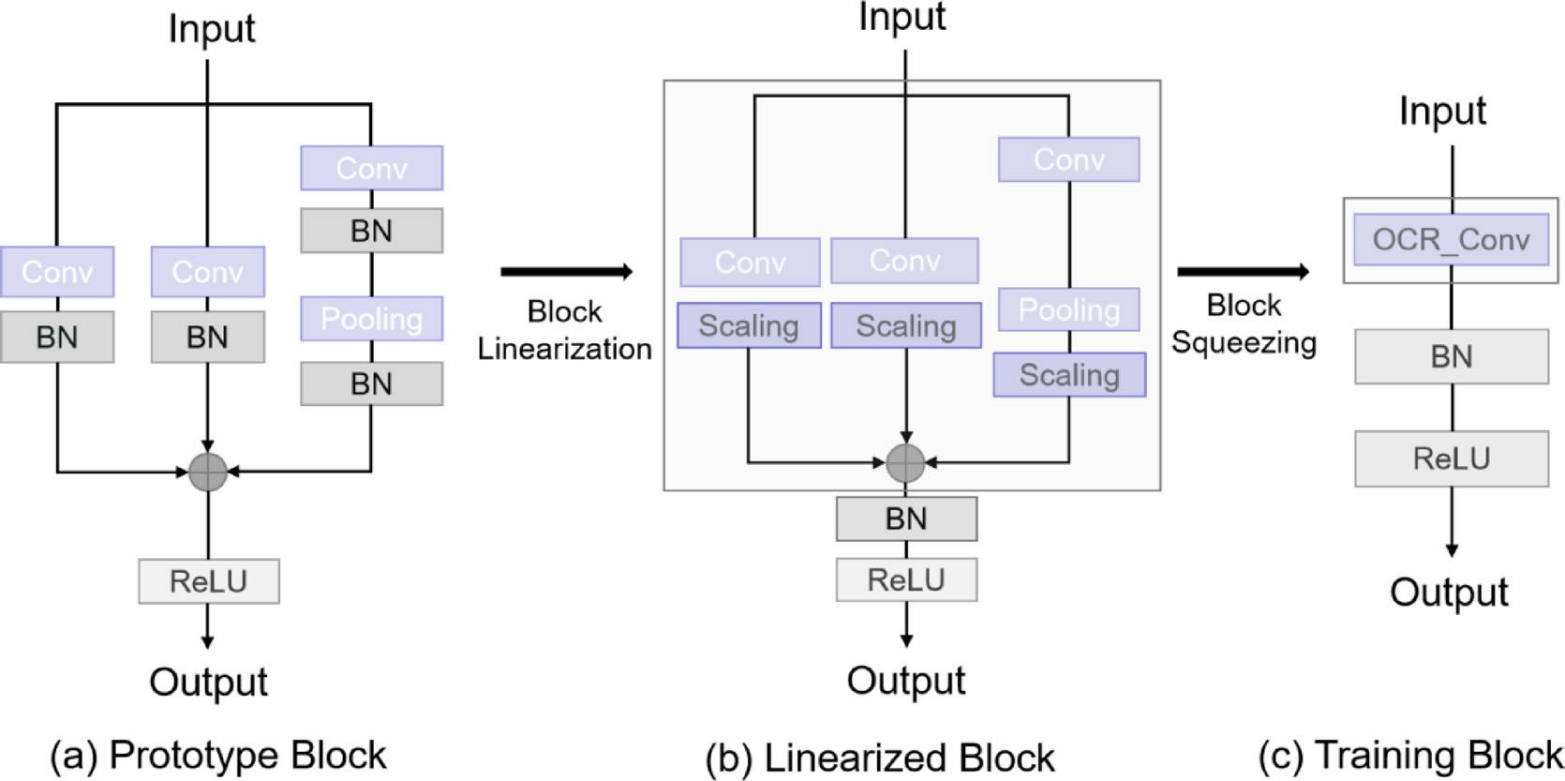
An overview of the proposed Online Re-Parameterization (OREPA), a two-stage pipeline. In the first stage (Block Linearization), we remove all the non-linear components in the prototype re-param block. In the second stage (Block Squeezing), we merge the block to a single convolutional layer (OCRConv). Through the steps, we significantly reduce the training cost while keep the high performance.

A standard convolution module usually consists of a batch normalization layer, regular convolution layer, and activation function. This configuration usually results in a large number of similar feature maps, which results in high computational cost and memory consumption. OCRConv is a convolutional layer built by the Online Convolutional Reparameterization (OREPA) technique^20^, which aims to optimize memory usage and computational efficiency during model training to enhance the overall performance and resource utilization of convolutional neural networks. Therefore, we introduce the Online Convolutional Reparameterization (OREPA) technique into the convolution of the YOLOv10 network to reduce the model complexity while improving the training speed. The proposed technique mainly uses two key technical steps as shown in Fig. 2: Block Linearization and Block Squeezing. Firstly, in the Block Linearization stage, OREPA removes the nonlinear layers commonly used in the training process. Although these layers help to improve the expressive power of the model, they increase the computational complexity and memory consumption. Instead, a linear scaling layer is introduced, which is a key component of the OREPA technique. This layer scales feature maps using learnable vectors, enabling more efficient weight updates during training. By maintaining the diversity of optimization directions and feature expressiveness, this approach not only improves model performance but also reduces training costs and memory usage. Next, in the block-squeezing stage, OREPA merges the linearized blocks into a single convolutional layer. This means that the original complex architecture consisting of multiple convolutional layers and batch normalization layers will be simplified into a single convolutional layer, a process that greatly reduces the complexity and memory requirements at training time. Through block squeezing, OREPA reduces the computation and storage overhead of intermediate feature maps, thus improving the training efficiency. Specifically, block extrusion represents all linear layers in the parameter-heavy structure as convolutional layers with corresponding parameters, and compresses multiple layers and multiple branches into a single convolutional layer, which simplifies the model structure and reduces the consumption of computational resources.

### SPCC

The C2f module makes it difficult to locate dense small targets under complex backgrounds. with the increasing depth of convolutional neural networks (CNNs), repeated convolution operations tend to induce feature redundancy and similarity across channels., which means there is a lot of repeated information in the deep feature map, resulting in information redundancy and excessive model parameters.

To address this bottleneck, partial convolution (PConv) introduced in the FasterNet framework^21^ achieves efficient feature extraction through structured sparsity: Given an input feature map h×w×c, PConv applies convolutional transformations exclusively to a selected channel subset $h \times w \times c_{p}^{2}$. The processed feature tensor is subsequently concatenated with the original unprocessed channels along the channel dimension while preserving the output dimensionality. This design maintains complete channel information while reducing floating-point operations of conventional convolution, significantly enhancing computational efficiency.

For enhanced multi-scale feature discriminability, the Shuffle Attention^43^ (SA) mechanism partitions input feature channels into multiple mutually exclusive subgroups. Within each subgroup, dual-path attention modeling operates in parallel: the channel attention branch generates channel-wise weight vectors via global average pooling, while the spatial attention branch constructs spatial saliency maps through group normalization. Attention weights fused by element-wise multiplication are applied to corresponding sub-features, with cross-subgroup feature interaction achieved through differentiable channel shuffling. This architecture enables more efficient capture of complex patterns and structures within feature maps while circumventing redundant computations without compromising representational capacity.

**Fig. 3:**
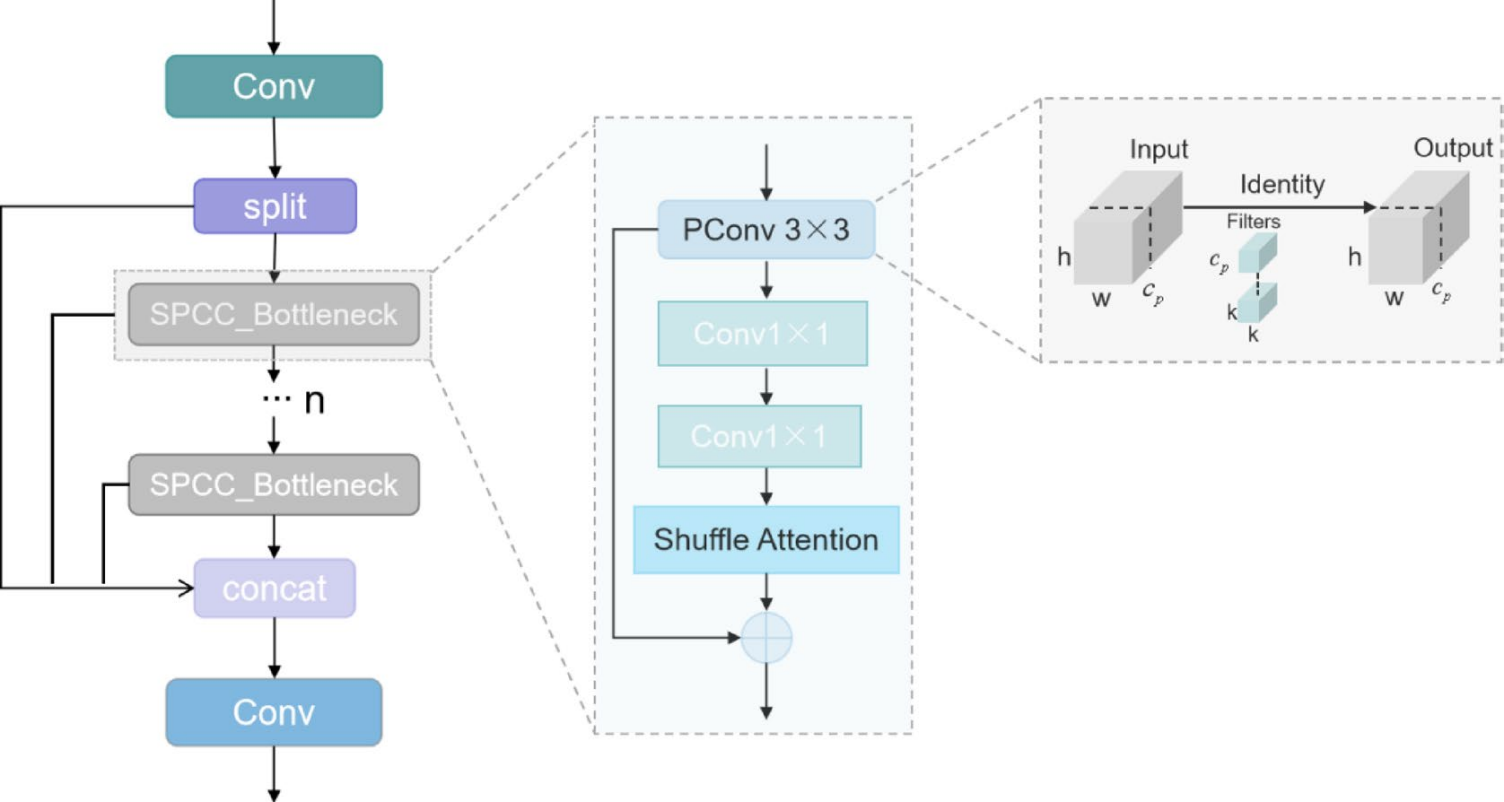
The structure of SPCC (mainly shows the SPCC_BottleNeck of the SPCC module designed in this paper).

Building upon these advances, we propose the SPCC_Bottleneck to reconfigure the C2f module in OSD-YOLO, This is shown in Fig. 3: Stage 1 harnesses PConv for selective feature extraction to capture fine-grained local details; Stage 2 executes cross-channel integration via 1 × 1 standard convolution; Stage 3 incorporates SA for dual-dimensional (channel-space) dynamic feature calibration. This tri-stage cascade synergistically reduces model complexity while enhancing backbone network functionality through multi-scale feature refinement, thereby establishing discriminative feature representations within constrained computational resources.

### DySample

**Fig. 4:**
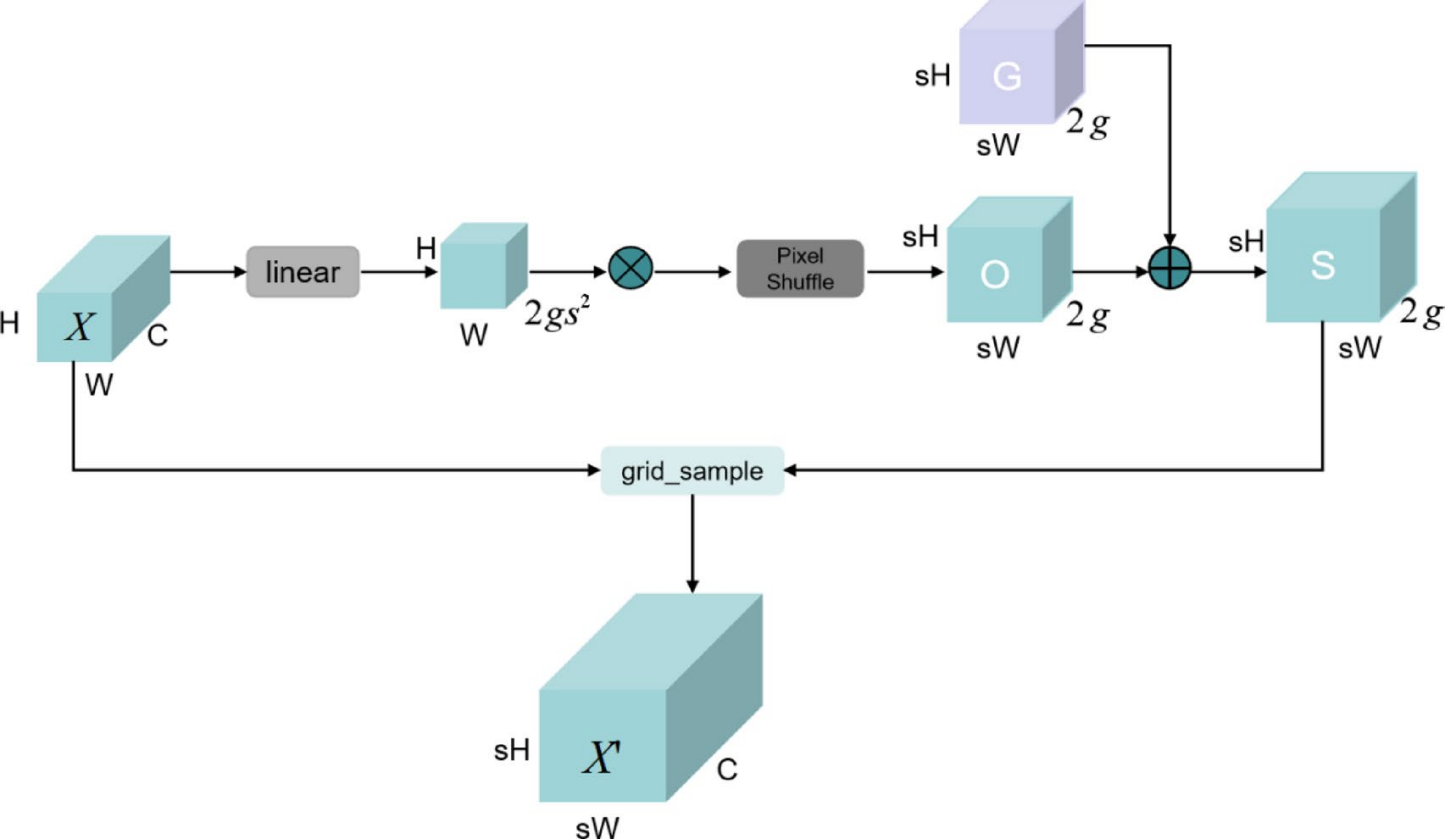
The DySample implementation diagram.The linear layer and pixel shuffle technique are combined with a fixed range factor to generate the offset (O), which is then added to the original grid position (G) to obtain the sampling set (S). The input features are resampled using the grid_sample function to obtain the upsampled features (X’).

The YOLOv10n model typically employs nearest-neighbor interpolation for its upsampling layer, a method that simply duplicates adjacent pixel values. However, this approach presents significant limitations: it ignores smooth transitions between pixels, uses only a few surrounding pixels for prediction, and focuses mainly on spatial information while ignoring the semantic information of the feature map; it is prone to blur and distortion when dealing with complex scenes or edge details, and performs especially poorly when dealing with small targets. In addition, the traditional kernel-based upsampling process requires a large amount of computation and parameter overhead, which is not conducive to the realization of lightweight network architectures. Therefore, the DySample^22^ module is introduced in the feature fusion step to replace the original upsampling module. DySample realizes content-aware upsampling by dynamically determining the optimal sampling position of each output pixel through a learning mechanism, which not only improves the quality and flexibility of upsampling but also recovers small targets and edge information more accurately. In addition, DySample reduces model complexity and computational cost while maintaining high performance by reducing the need for high-resolution feature inputs and optimizing the computational process. The DySample flowchart is shown in Fig. 4.

Given an input feature map ${\text{X}}$ of size $C \times H \times W$ and a point sampling set S of $2g \times sH \times sW$, where $2g$ denotes the X and y coordinates. DySample first generates offsets of size $2g{{\text{s}}^2} \times H \times W$ through a 1 × 1 convolutional layer (linear projection layer) with input and output channels C and $2g{s^2}$. Then, it is reshaped into an offset O of $2g \times sH \times sW$ by the Pixel Shuffle operation, while the sampling set S is the sum of the offset O and the original sampling grid G. Finally, the grid_sample function resamples the input feature map X using the positions in the point sampling set S to produce a feature map $X'$ of size $C \times sH \times sW$. The specific implementation formula is as follows:

1 $$\text{O}=\text{linear(X)}$$

2 $$\text{S = G + O}$$

3 $$\text{X}^{\prime}=\text{grid}\_\text{sample(X, S)}$$

### DFMA

The self-attention mechanism is extensively utilized in object detection due to its strong global modeling capability and ability to capture long-range dependencies. In wide-area images captured at high altitudes, small vehicle targets often occupy only a few pixels, making them challenging to detect. To place greater emphasis on small target regions within images during object detection, this study proposes an efficient DFMA attention mechanism.

**Fig. 5:**
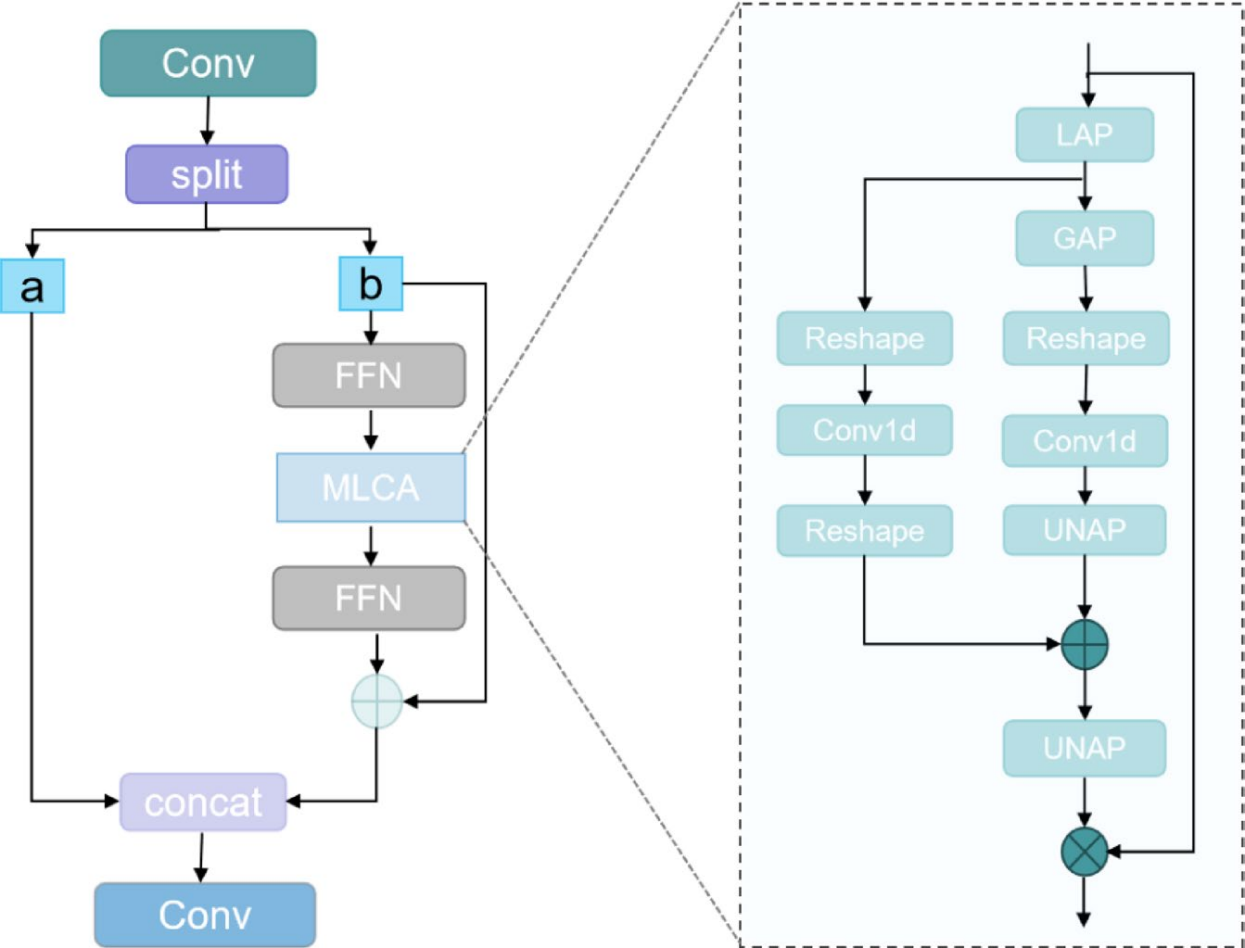
The structure of DFMA(mainly showing the design of the joined MLCA module and the principle of DFMA implementation).

The DualFeed-MixAttention Network (DFMA) is a novel spatial attention architecture that comprises two feed-forward neural networks (FFNs) and an innovative Mixed Local Channel-Spatial Attention (MLCA)^44^ module. The design aims to optimize semantic perception in complex scenes through multi-scale feature modeling and dynamic feature recalibration. As shown in Fig. 5, the input feature map is first decomposed into two parallel branches, a and b, by a 1 × 1 convolutional layer. Branch a retains the original semantic information to maintain the base feature flow, while branch b performs in-depth feature modulation by cascading a two-stage feed-forward neural network (FFN) and an MLCA module. The FFN enhances the model’s ability to capture complex patterns through nonlinear transformations, and the MLCA module integrates local and global information extraction units and channel-space fusion units. The former uses local average pooling (LAP) and global average pooling (GAP) to extract detail-sensitive features and global structural priors from the feature map, respectively. The latter models long-range dependencies between channels using 1D convolution (Conv1D) and combines it with deformable convolution to generate spatial attention masks. Ultimately, it realizes dynamic feature recalibration through weight fusion (critical feature enhancement and noncritical feature suppression). After the two-branch features are spliced together, a 1 × 1 convolutional layer accomplishes cross-channel information fusion, which further enhances the model’s characterization capability. The DFMA module is typically placed in the final layer of the backbone network, where the resolution is relatively low. This effectively alleviates quadratic computational complexity. After being integrated into detection frameworks such as YOLO with low computational cost, the proposed architecture significantly improves the identifiability of small target features and the robustness of detection in complex scenes.

### Double small target detection network

The YOLOv10 classification head uses a lightweight architecture of two 3 × 3 depthwise separable convolutional layers and one 1 × 1 convolutional layer. The original YOLOv10 detection head is designed based on multi-scale feature maps. When the input resolution is 640 × 640, the backbone network generates three-level feature maps of 80 × 80 (level P3), 40 × 40 (level P4), and 20 × 20 (level P5) through progressive downsampling with a step size of 2. These correspond to the construction of small, medium, and large detection heads, respectively. The P3-level, high-resolution feature map (80 × 80) covers an 8 × 8 pixel area of the original image. It focuses on capturing detailed information to detect small objects larger than 16 × 16 pixels. Conversely, the P5-level, low-resolution feature map (20 × 20) covers an area of 32 × 32 pixels per grid. It relies on global semantic information to detect large objects larger than 64 × 64 pixels. In the UAV image scene, however, targets are usually distributed at a very low pixel density. For example, targets are usually 4 × 4 pixels. This makes the P5-level feature map dilute small target features due to its large grid coverage (32 × 32 pixels). In contrast, the P3-level feature map can retain more detailed information due to the lack of high-level semantic association. Consequently, the missed detection rate of small targets increases.

To address the issue of bottlenecks, we propose a design that incorporates dual small object detection layers. Within the neck network, the first detection layer leverages the feature map from the P2 level to create an x-small detection head, which is responsible for detecting objects larger than 4 × 4 pixels. The second detection layer integrates the feature maps from both the P1 and P2 levels, utilizing cross-level feature enhancement to construct the xx-small detection head, specifically targeting ultra-small objects that are larger than 2 × 2 pixels. This design fully capitalizes on the benefits of shallow, high-resolution feature maps while mitigating information attenuation commonly caused by deeper convolutions, thanks to the complementary features between layers. This approach significantly enhances the model’s capability to represent small targets. To further optimize deployment efficiency, this paper reconstructs the detection network architecture, retaining only the xx-small, x-small, small, and medium detection heads. Consequently, the reconstructed dual small object detection network achieves a more balanced trade-off between model complexity and accuracy.

## Experiments and results

### Experimental environment and parameter configuration

The training and development of this experiment is based on Pytorch framework under Linux server, using Ubuntu system, the specific experimental environment is shown in Table 1.

**Table 1:** Experimental environment configuration.

System name	Environmental parameters
Operating system CPU GPU Python CUDA Pytorch	Windows 11 AMD Ryzen9 7945HX NVIDIA GeForce RTX 4090 3.9 12.1 2.4.1

The experiments were trained, validated, and tested in the same environment. All training was done from scratch, initialized with weights trained on the COCO dataset. Experimental hyperparameters: the input image was resized to 640 × 640 pixels; momentum was set to 0.937, batch size to 16, and weight decay to 0.0005; the number of epochs was 200.The optimizer was Stochastic Gradient Descent (SGD), with an initial learning rate of 0.01. and the rest of the hyperparameters were taken as defaults from YOLOv10.

### Description of the datasets

To confirm the effectiveness of the improved OSD-YOLOv10 model, the public datasets VisDrone2019^45^ and UAVDT^46^ are selected for experiments in this paper.

The VisDrone2019 dataset is a large UAV vision dataset open-sourced by Tianjin University. It contains 10,209 still images taken by UAVs from different angles, of which 6,471 are the training set, 548 are the validation set, and 1,610 are the test set, totaling about 2.6 million target instances covering 10 target categories such as pedestrians, cars, and trucks, and covering 14 urban and rural environments in China.

The UAVDT dataset consists of 50 videos and 38,327 images containing three object classes: cars, buses, and trucks. Among them, 23,258 images were used for training and 15,069 images were used for testing.

**Fig. 6:**
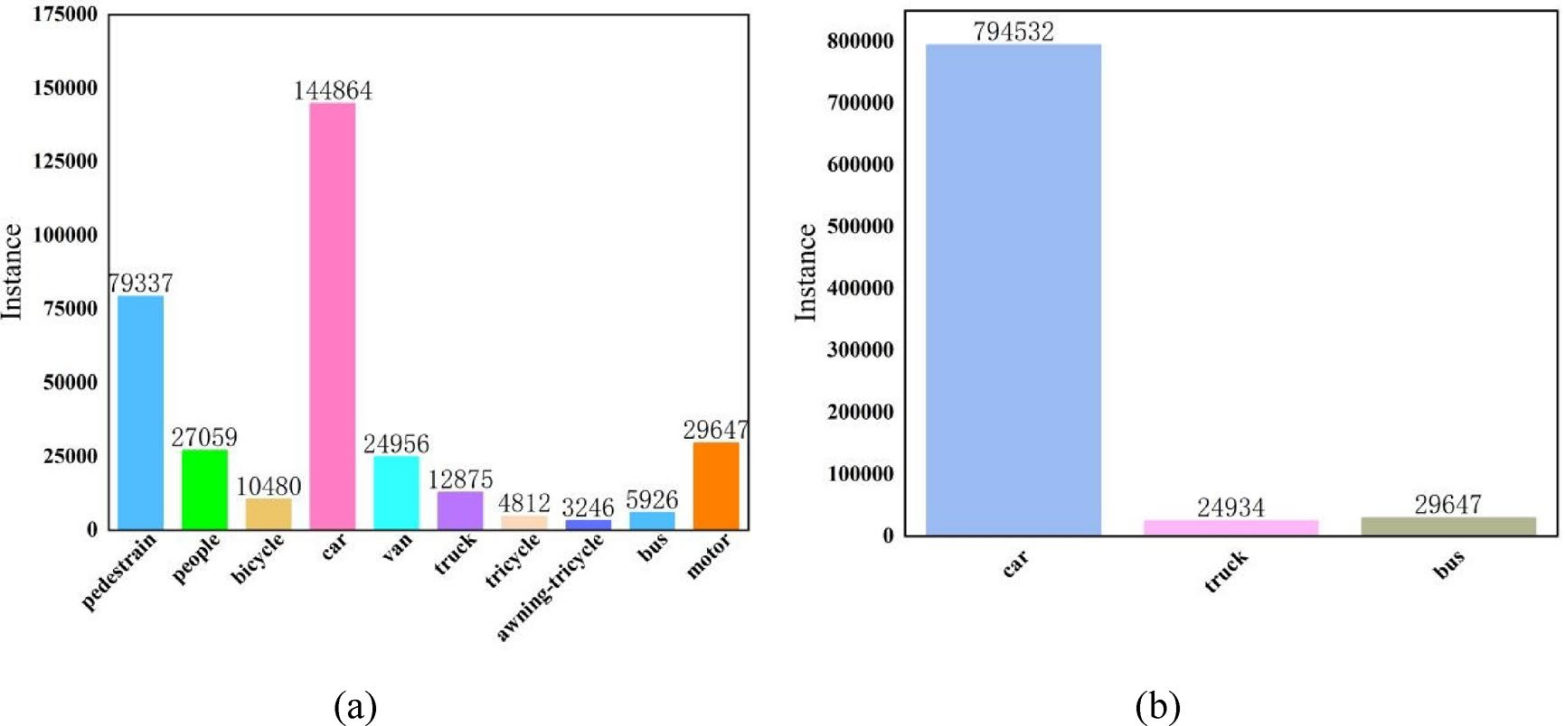
(**a**) Each category and quantity of the VidroneDET-2019 dataset. (**b**) Each category and number of UAVDT datasets.

The target types in both datasets are diverse and predominantly small, and Fig. 6 shows the percentage of targets in each category.

### Evaluation criteria

The model was evaluated based on its detection performance and model size. Precision (P), Recall (R), Average Precision (AP), and Mean Average Precision (mAP) were used to measure the accuracy of all object categories. In addition, the computational complexity of the network was evaluated using the metric gigafloating point operations per second (GFLOPs). The size of the model was determined by the number of parameters, and the real-time processing speed of the model was measured in frames per second (FPS). mAP@0.5 and mAP@0.5:0.95 represent the mean AP for each class when the Intersection over Union (IoU) threshold is 0.5 and when IoU ranges from 0.5 to 0.95, respectively, where IoU is the overlap rate between the predicted bounding box and the true bounding box, i.e. the ratio of intersection and union, which is used to evaluate the accuracy of object detection.

True positives (TP) are cases where both the actual label and the prediction are positive. A false negative (FN) occurs when the label is positive but the prediction is falsely negative. A false positive (FP) occurs when the actual label is negative but the prediction is positive. True Negative (TN) indicates a situation where both the label and the prediction are negative.The formulas for P and R are given below:

4 $$\text{P}=\frac{\text{TP}}{\text{TP + FP}}$$

5 $$\text{R}=\frac{\text{TP}}{\text{TP + FN}}$$

AP is the area under the PR curve; the higher the AP, the higher the accuracy. mAP is the average of the APs of all categories; a higher mAP indicates better model performance. the formulas for AP and mAP are given below:

6 $$\text{AP}=\int_{0}^{1} \text{P(R)dR}$$

7 $$\text{mAP}=\frac{1}{\text{n}}\sum\limits_{{i=1}}^{\text{n}} {\text{AP(i)}}$$

where n is the number of instances of a given category and AP(i) is the accuracy of detection of category i.

GFLOPs denotes the number of billion floating point operations per second, which is used to measure the computational complexity of the model.GFLOPs is calculated as follows:

8 $$\text{GFLOPs}=\frac{{\text{TotalFloatingPointOperations}}}{{{{10}^9}}}$$

The higher the GFLOPs, the higher the computation and hardware requirements.

FPS is an important indicator of the model’s image processing speed. The higher the FPS value, the more images the model processes per unit time, which means the faster the recognition speed. The calculation formula is as follows:

9 $$\text{FPS}=\frac{{\text{FrameNum}}}{{\text{ElapsedTime}}}$$

Where FrameNum is the total number of frames detected and ElapsedTime is the total time taken by the model to perform the detection.

### Ablation experiment

To demonstrate the effectiveness of the proposed OSD-YOLOv10 network, we performed ablation experiments based on the baseline network on the VisDrone2019 dataset. Given the inherent randomness in deep learning experiments, all results reported in this study are averaged over multiple runs to ensure robustness and reliability. The experiments first evaluate the performance of each module individually within the baseline network. Subsequently, a stacking approach is employed to incrementally integrate modules into the baseline network, allowing for a comparative analysis of their effectiveness, as shown in Table 2.

**Table 2:**
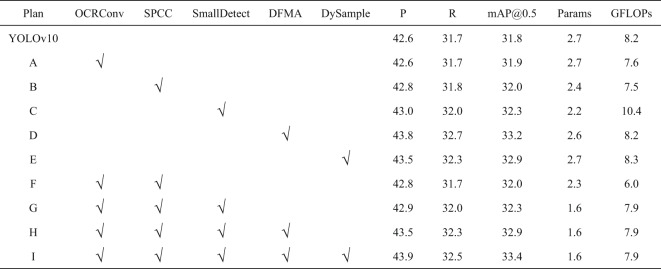
Ablation effects of each Module.

A (OCRConv): The OCRConv module, constructed using the online convolutional reparameterization technique, replaces standard convolutions. By removing nonlinear layers and merging convolutional layers, this approach simplifies the model architecture and reduces computational resource consumption. Resulting in a 7.3% reduction in computational load compared to the original model.

B (SPCC): The lightweight SPCC structure improves the C2f module by incorporating partial convolution operations and the efficient Shuffle Attention mechanism. This design reduces information redundancy and model parameter counts without compromising performance. Results show an 11.1% reduction in computation and an 8.1% reduction in parameters while maintaining precision and recall rates.

C (Dual Small Target Detection Layer): The small target detection layer is reconstructed by combining the shallowest (P1) and shallow (P2) features, enhancing the model’s ability to detect small targets. This modification reduces the model’s parameter count by 18.5% while slightly improving accuracy.

D (DFMA): The original PSA module is replaced with the more efficient DFMA module, which utilizes a hybrid local-channel attention mechanism (MLCA). This lightweight and efficient module improves precision, recall, and mAP@0.5 by 1.2, 1.0, and 1.4% points, respectively, while keeping parameter counts and GFLOPs stable.

E (DySample): The dynamic upsampling module, DySample, enhances the neck network’s feature fusion capability. Compared to the original model, DySample improves precision, recall, and mAP@0.5 by 0.9, 0.6, and 1.1% points, respectively.

F (A + SPCC): Combining the OCRConv module (A) with the SPCC module (B) further reduces model complexity while slightly improving precision.

G (F + Small Target Detection Layer): Reconstructing the small target detection layer on top of Module F significantly reduces the model’s parameter count to only 40.7% of the original model, with a slight improvement in precision and recall.

H (G + DFMA): Adding the DFMA module to Module G improves precision, recall, and mAP@0.5 compared to using Module G alone, achieving better detection performance without increasing complexity.

I (H + DySample): Incorporating DySample into Module H further enhances precision, recall, and mAP@0.5 while maintaining lower computational complexity and parameter counts compared to the original model.

The final configuration (I) achieves an optimal balance between high accuracy and lightweight design, making it well-suited for scenarios requiring both high precision and real-time performance.

### Comparative with multiple datasets

The use of different datasets for training and validation plays a key role in the performance and robustness of the model. It can significantly improve the generalization ability, performance, and robustness of object detection models, ensuring their effectiveness in a variety of practical applications. In this paper, the original model YOLOv10n and the improved model OSD-YOLOv10 are tested on two datasets, VisDrone2019 and UAVDT, respectively, and the experimental results are shown in Tables 3 and 4 below.

**Table 3:** Comparison experiment of different categories in the visdrone dataset.

Class	YOLOv10n				OSD-YOLOv10			
	*P*	*R*	mAP@0.5	mAP@0.5:0.95	*P*	*R*	mAP@0.5	mAP@0.5:0.95
all	42.6	31.7	31.8	18.2	43.9	32.5	43.4	19.1
pedestrain	41.3	34.4	33.6	14.3	41.4	34.4	33.7	14.3
people	47.8	25.3	27.8	10.3	48.5	25.3	28	10.4
bicycle	22	11.7	9.12	3.66	22.9	11.8	9.19	3.69
car	63.8	74.6	75.1	52	63.9	75	75.9	52
van	47.1	34.5	36.3	25	47.1	34.4	36.3	25
truck	43.1	26.7	27.8	17.2	43.2	26.6	27.8	17.2
tricycle	37.1	21.9	19.8	10.3	37.8	22.1	20.6	10.5
awning-tricycle	25.3	12.2	11.2	7.18	25.6	12.5	11.8	7.21
bus	55.9	38.6	42.7	29	55.9	38.6	42.6	28.8
motor	44.6	39.3	36.7	15.1	44.9	39.3	15.5	45

**Table 4:** Comparison experiment of different categories in the UAVDT dataset.

Class	YOLOv10n				OSD-YOLOv10			
	*P*	*R*	mAP@0.5	mAP@0.5:0.95	*P*	*R*	mAP@0.5	mAP@0.5:0.95
all	41.8	32.9	30.9	17.6	42.3	33.1	31.5	17.8
car	80.2	62.1	69.7	39.3	80.3	62.1	69.9	39.3
truck	21.5	10.6	7.99	46.6	21.9	10.6	8.01	46.8
bus	21.3	25.5	12.3	7.9	21.3	25.6	12.6	8

As shown in Table 3, the OSD-YOLOv10 model demonstrates enhanced detection performance with precision and mean average precision (mAP) improvements of 1.3% and 1.6%, respectively. Analyzing the accuracy performance of each category, we can see that the OSD-YOLOv10 model has better detection performance in detecting small targets such as people, bicycles, tricycles, and motors, which are increased by 0.7%, 0.9%, 0.7%, and 0.3% respectively compared to YOLOv10. The results demonstrate the architectural advantages of OSD-YOLOv10 in detecting small objects.

Comparison with Table 4 shows that the Mean Average Precision (mAP) of the OSD-YOLOv10 model is increased by 0.6% compared to the YOLOv10 model, and the accuracy of each category shows a small improvement. The results show that OSD-YOLOv10 also performs better than YOLOv10 on the UAVDT dataset.

**Fig. 7:**
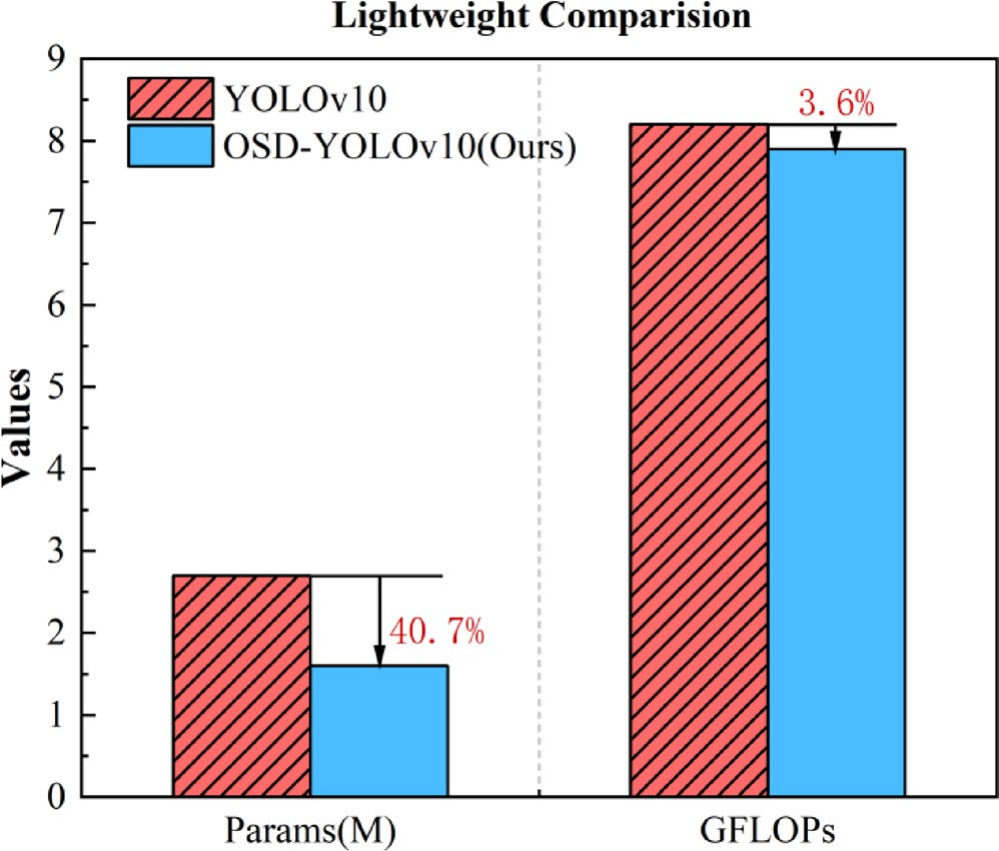
Lightweight comparison of the OSD-YOLOv10 and YOLOv10 models in the VisDrone dataset and UAVDT dataset.

It is important to prioritize lightweighting the model while maintaining accuracy to facilitate model deployment on resource-constrained edge devices, thereby improving the detection speed at the edge and achieving real-time detection. As shown in Fig. 7, a comparison was made between OSD-YOLOv10 and YOLOv10 in terms of parameters and GFLOPs.The improved OSD-YOLOv10 model exhibited a reduction of 40.7% in the number of parameters and 3.6% in GFLOPs. In summary, the OSD-YOLOv10 model underwent further optimization while maintaining the high accuracy of the original model and achieved a significant reduction in model size, which is beneficial for subsequent deployment on the edge for real-time detection.

### Comparative with different models

To evaluate OSD-YOLOv10’s superiority, we conducted a large number of comparative experiments for validation. Table 5 compares OSD-YOLOv10 and other models on the VisDrone2019 dataset. The selection criteria for the comparison algorithms were the number of parameters and computational complexity within a reasonable range.

We retained and emphasized the best performance results for each metric. As shown in Table 5, OSD-YOLOv10 has higher performance compared with the baseline model YOLOv10n, with 0.5% improvement in accuracy and 0.2% improvement in mAP@0.5. In addition, the number of parameters of OSD-YOLOv10 is reduced by about 1.1 M, and the computational complexity is reduced by 0.3GFLOPs. Compared with the typical lightweight models of the YOLO series, YOLOX-tiny, YOLOv5n, YOLOv7-tiny, and YOLOv8n, the accuracy is increased by 0.8%, 5.2%, 0.3%, and 1.1%, respectively, and is increased by 1.8%, 7.2%, 0.9%, and 1.3%, respectively, on mAP@0.5. Compared with YOLOX-tiny, YOLOv5n, YOLOv7-tiny, and YOLOv8n, the number of model parameters is reduced by 68%, 11.1%, 73.3%, and 46.7%, respectively. The computational complexity of the model is reduced by 48.3%, 40.2%, and 2.5%, respectively. Compared with recent research results, our model has better precision, recall, and mAP@0.5 than EfficientDet-D0^47^, Gold-YOLO^48^, and FFCA-YOLO^40^. It also has a lower number of parameters and computational complexity.In addition, compared with the recently released YOLOv11n^49^the precision, recall, and mAP@0.5 are increased by 0.5%, 0.2%, and 0.5%, respectively, and the parameter count of OSD-YOLOv10 is reduced by 38.5%.

**Table 5:** Comparison experiment of different model in the visdrone dataset.

Model	*P*	*R*	mAP@0.5	Params	GFLOPs
YOLOX-tiny	43.1	31.1	31.6	5	15.3
YOLOv5n	38.7	27.2	26.2	1.8	4.2
YOLOv7-tiny	43.6	31.6	32.5	6	13.2
YOLOv8n	42.8	31.6	32.1	3	8.1
YOLOv10n	42.6	31.7	31.8	2.7	8.2
EfficientDet-D0	40.1	29.1	30.4	3.9	2.5
Gold-YOLO	41	31.3	32.7	5.6	12.1
FFCA-YOLO	42.9	31.6	33.1	7.1	14.6
YOLOv11n	43.4	32.3	32.9	2.6	6.3
OSD-YOLOv10	43.9	32.5	33.4	1.6	7.9

**Fig. 8:**
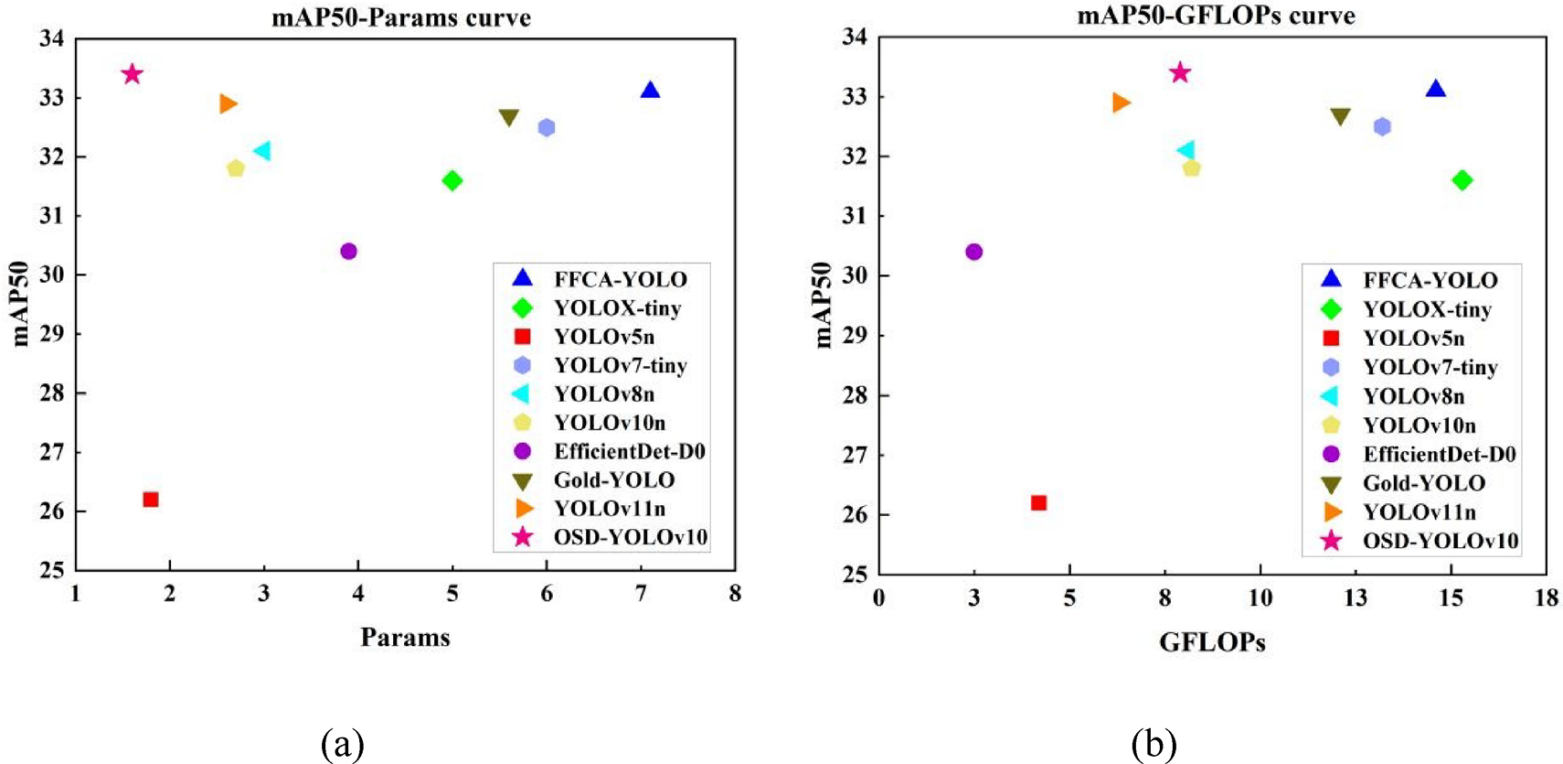
mAP50-Params (**a**) and mAP50-GFLOPs (**b**) scatter diagram of models on VisDrone2019 dataset.

Figure 8 shows the model mAP@0.5 versus the number of parameters and the computational complexity. The model in the upper left corner of the scatter plot is better. OSD-YOLOv10 achieves a better balance between accuracy, number of parameters, and computational complexity. OSD-YOLOv10 achieves higher mAP@0.5 and lower parameter count and computational complexity compared to other models.

**Table 6:** Comparison of processing speed of different models on VisDrone2019 dataset.

Model	FPS
Gold-YOLO	112
FFCA-YOLO	89
YOLOv11n	163
OSD-YOLOv10	136

Table 6 gives the processing speeds of OSD-YOLOv10 and the other three models with the highest detection accuracy. On the VisDrone2019 dataset, the FPS of OSD-YOLOv10 is 136. the processing speed is 27 frames per second slower compared to YOLOv11n. However, OSD-YOLOv10 has higher detection accuracy and a lower off-target rate. Additionally, OSD-YOLO outperforms Gold-YOLO and FFCA-YOLO by 24 and 47 frames per second, respectively. When detection accuracy is similar, OSD-YOLOv10 has a faster detection speed and requires fewer computations.

In summary, OSD-YOLOv10 achieves the highest mAP and lower computational complexity at a higher inference speed, representing the best configuration in terms of overall performance. This configuration illustrates the great potential and wide applicability of the scheme in practical applications to meet the requirements of high accuracy, low computational resources, and high real-time performance.

### Visual comparative analysis

To intuitively and efficiently demonstrate the detection efficacy of our proposed model, we conducted comparative analyses of model performance through confusion matrices and inference result visualizations.

**Fig. 9:**
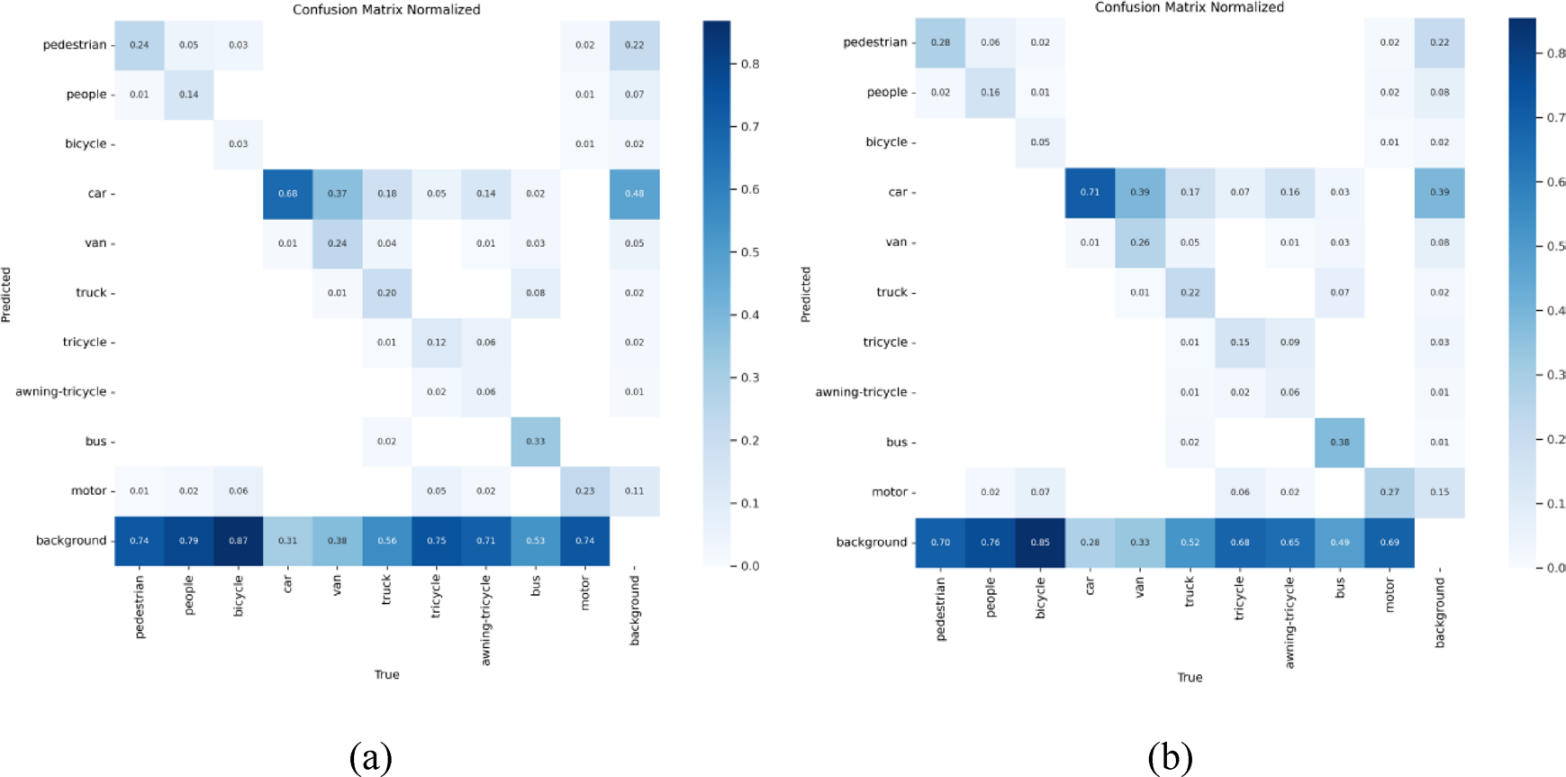
(**a**) Confusion matrix plot of YOLOv10; (**b**) Confusion matrix plot of OSD-YOLOv10.

To visualize the model’s capability in predicting target categories, we present the confusion matrices of YOLOv10 and our OSD-YOLOv10 in Fig. 9. The rows and columns represent the true and predicted categories, respectively. Values along the diagonal indicate the proportion of correctly predicted categories, while off-diagonal entries denote misclassification rates. As shown in Fig. 9, OSD-YOLOv10 exhibits darker diagonal coloration than YOLOv10, indicating enhanced prediction accuracy. However, for small objects (e.g., bicycles, tricycles, and motorcycles), a higher proportion of background misclassification persists, suggesting substantial missed detections. Although our improved model reduces the missed detection rate for these categories, their correct prediction ratios remain suboptimal. This limitation arises because bicycles—being compact transportation tools—typically appear in densely occluded formations, complicating reliable detection in complex backgrounds.

**Fig. 10:**
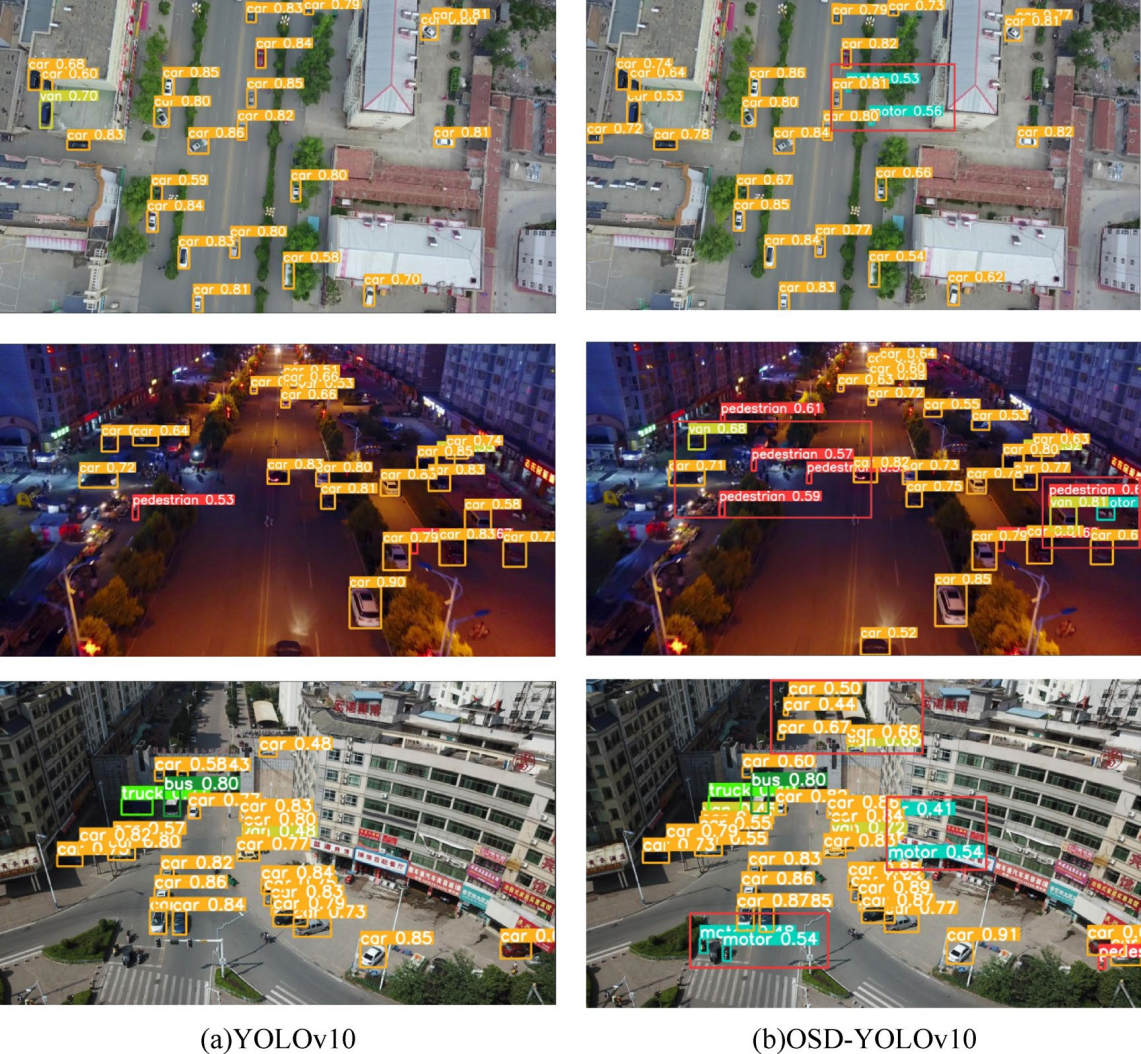
Visualization results between YOLOv10 (**a**) and OSD-YOLOv10 (**b**) on the VisDrone2019 dataset. (The experimental images are sourced from the VisDrone2019 dataset.).

To visually validate the detection performance, we performed inference experiments comparing YOLOv10 and OSD-YOLOv10 across multiple representative scenarios. These scenes contain diverse small objects well-suited for evaluation. Detection results are illustrated in Fig. 10, with key regions magnified for visual clarity. Comparative analysis reveals that our method achieves superior accuracy for distant targets within the field of view relative to YOLOv10. Furthermore, it effectively mitigates prevalent false positives and missed detections of small objects under UAV perspectives, thereby significantly enhancing overall detection performance.

## Conclusions

In this paper, we propose OSD-YOLOv10, a lightweight and efficient vehicle target detection model tailored for UAV aerial photography, addressing the challenge of achieving efficient deployment on resource-constrained devices such as UAVs. To realize a lightweight architecture, we introduce the SPCC module to replace the C2f module in the original network.The SPCC_Bottleneck structure ensures robust feature extraction while significantly reducing model computation and parameter counts.Additionally, we employ the online convolutional reparameterization technique to eliminate nonlinear layers and merge convolutional layers, simplifying the model structure and minimizing computational resource consumption. However, lightweight operations may result in information loss. To mitigate this, we propose an efficient spatial attention mechanism, DFMA, which enhances the model’s ability to represent features of vehicles across varying scales. DFMA leverages a dual-layer feed-forward neural network and a hybrid local-channel attention module, combined with a reconfigured dual-layer small-target detection network, to improve recognition accuracy for small targets in complex environments. Furthermore, we introduce the DySample module, which achieves content-aware upsampling by dynamically determining the optimal sampling location for each output pixel. This approach enables more accurate recovery of small targets and edge details, reducing model complexity and computational costs while maintaining high performance. Finally, experiments on VisDrone2019 and UAVDT datasets validate the higher detection accuracy and lower computational complexity of OSD-YOLOv10 compared to other YOLO series models and lightweight models, proving that it achieves an optimal balance between high accuracy and low resource consumption, and is suitable to be deployed in UAV airborne hardware to carry out vehicle target detection.

## Data Availability

The datasets used and analysed during the current study available from the corresponding author on reasonable request.
